# Identifying new biomarkers and potential therapeutic targets for breast cancer through the integration of human plasma proteomics: a Mendelian randomization study and colocalization analysis

**DOI:** 10.3389/fendo.2024.1449668

**Published:** 2024-09-16

**Authors:** Jingshuang Song, Huawei Yang

**Affiliations:** ^1^ Department of Breast Surgery, Guangxi Medical University Cancer Hospital, Nanning, China; ^2^ Department of Breast and Thyroid Surgery, Affiliated Hospital of Guilin Medical University, Guilin, China; ^3^ Laboratory of Breast Cancer Diagnosis and Treatment Research of Guangxi Department of Education, Guangxi Medical University, Nanning, China

**Keywords:** breast cancer, proteomics, biomarkers, drug targets, Mendelian randomization, colocalization analysis

## Abstract

**Background:**

The proteome is a crucial reservoir of targets for cancer treatment. While some targeted therapies have been developed, there are still significant challenges in early diagnosis and treatment, highlighting the need to identify new biomarkers and therapeutic targets for breast cancer. Therefore, we conducted a comprehensive proteome-wide Mendelian randomization (MR) study to identify novel biomarkers and potential therapeutic targets for breast cancer.

**Methods:**

Protein quantitative trait locus (pQTL) data were extracted from two published plasma proteome-wide association studies. Genetic variants associated with breast cancer were obtained from the Breast Cancer Association Consortium, which included 133,384 cases and 113,789 controls, and the Finnish cohort study, comprising 18,786 cases and 182,927 controls. We employed summary-based MR and colocalization methods to identify potential drug targets for breast cancer, which were subsequently validated using a two-sample MR approach. Finally, a protein-protein interaction (PPI) network was constructed to detect interactions between the identified proteins and existing cancer drug targets.

**Results:**

Gene-predicted levels of ten proteins were associated with breast cancer risk. Decreased levels of CASP8, DDX58, CPNE1, ULK3, PARK7, and BTN2A1, as well as increased levels of TNFRSF9, TNXB, DNPH1, and TLR1, were linked to an elevated risk of breast cancer. Among these, CASP8 and DDX58 were supported by tier-one evidence, while CPNE1, ULK3, PARK7, and TNFRSF9 received tier-two evidence support. The remaining proteins, TNXB, BTN2A1, DNPH1, and TLR1, were supported by tier-three evidence. CASP8, DDX58, CPNE1, ULK3, PARK7, and TNFRSF9 have already been identified as targets in drug development and potential therapeutic targets for breast cancer treatment. Additionally, ULK3 showed promise as a prognostic biomarker for breast cancer.

**Conclusions:**

The present study identified several novel potential drug targets and biomarkers for breast cancer, providing new insights into its diagnosis and treatment. The integration of PPI and druggability evaluations enhances the prioritization of these therapeutic targets, paving the way for future drug development efforts.

## 1 Introduction

Breast cancer is one of the most prevalent malignancies among women worldwide, with an incidence that continues to rise. In 2023, the United States alone had some 300,000 new cases, accounting for approximately 15.32% of all newly diagnosed cancers. Simultaneously, around 43,000 deaths due to breast cancer were recorded, constituting 7.2% of all cancer-related mortalities. Breast cancer profoundly affects patients’ quality of life and overall health (1, 2), and despite the advancements in treatment modalities, significant challenges, including the inadequacy of early diagnosis, the unpredictability of treatment outcomes, and the development of drug resistance, remain (3). As a result, identifying novel biomarkers and therapeutic targets has become a critical focus of contemporary breast cancer research.

Proteomics is a high-throughput technology that can reflect normal physiological processes and cancer pathobiology. Researchers can discover novel cancer-associated biomarkers by analyzing protein expression profiles in tumor tissues or body fluids, offering theoretical support for personalized patient treatment (4). Previous observational studies have identified specific circulating proteins associated with breast cancer risk (5–8); however, reverse causality or confounding factors may obscure the conclusions drawn from traditional observational research.

Mendelian randomization (MR) is a method employed to estimate causal effects within specific hypothetical contexts based on the principle that genes are randomly assigned from parents to offspring during gametogenesis and conception. Unlike traditional observational studies, MR is not susceptible to the biases of reverse causality or confounding (9). Consequently, several studies using the MR approach have uncovered various circulating proteins linked to breast cancer risk. For example, Jia and colleagues employed a two-sample MR approach to assess the association between 1,142 proteins and breast cancer risk, identifying 22 proteins linked to this risk (10). Similarly, Shu et al. utilized the same method to examine 2,994 proteins, uncovering 56 associated with breast cancer risk (11). They further explored the relationship between 1,890 circulating proteins and various breast cancer subtypes, identifying 98 proteins significantly associated with one or more subtypes (12). Additionally, Mälarstig and colleagues used a two-sample MR approach to identify five proteins potentially causally linked to breast cancer (13). However, these studies often relied on a single analytical method or faced limitations in protein coverage and sample size, generating inconsistent findings that have hindered a comprehensive understanding of the relationship between protein expression and breast cancer risk. Two recent studies have further advanced this field by employing bidirectional MR and colocalization analyses to systematically explore potential drug targets between plasma proteins and breast cancer. Colocalization analysis effectively distinguishes causal relationships from linkage disequilibrium (LD) within the genome, thereby enhancing the reliability of the results. This multifaceted approach offers crucial insights into identifying potential drug targets for breast cancer and lays a solid foundation for future drug development (14, 15).

The present study utilized Summary-based Mendelian Randomization (SMR), an advanced extension of traditional MR methods. The SMR approach integrates independent genome-wide association study (GWAS) summary data with quantitative trait locus (QTL) data, thereby prioritizing potential causal genes identified in GWAS. Unlike conventional MR methods, SMR can more accurately distinguish potential causal associations from LD within the genome, yielding more reliable causal inferences (16). By combining SMR with colocalization analysis, we systematically investigated the relationship between the human plasma proteome and breast cancer risk. This innovative approach enabled us to address some of the limitations of previous studies, providing more robust supporting evidence (17). Given the limitations of evidence from a single methodological approach, we further employed a two-sample MR approach for validation, systematically assessing the potential of proteins as novel biomarkers and therapeutic targets for breast cancer. Future research should integrate multi-omics data, including expression quantitative trait locus (eQTL) and methylation quantitative trait locus (mQTL), by combining SMR and two-sample MR methods, as such an approach could offer new perspectives on the molecular mechanisms of breast cancer and provide critical insights for identifying targets in personalized therapy.

## 2 Materials and methods

The present research adhered to the guidelines outlined in the Strengthening the Reporting of Observational Studies in Epidemiology (STROBE) (Supplementary STROBE-MR checklist table) (18).

### 2.1 Study design

**Figure 1:**
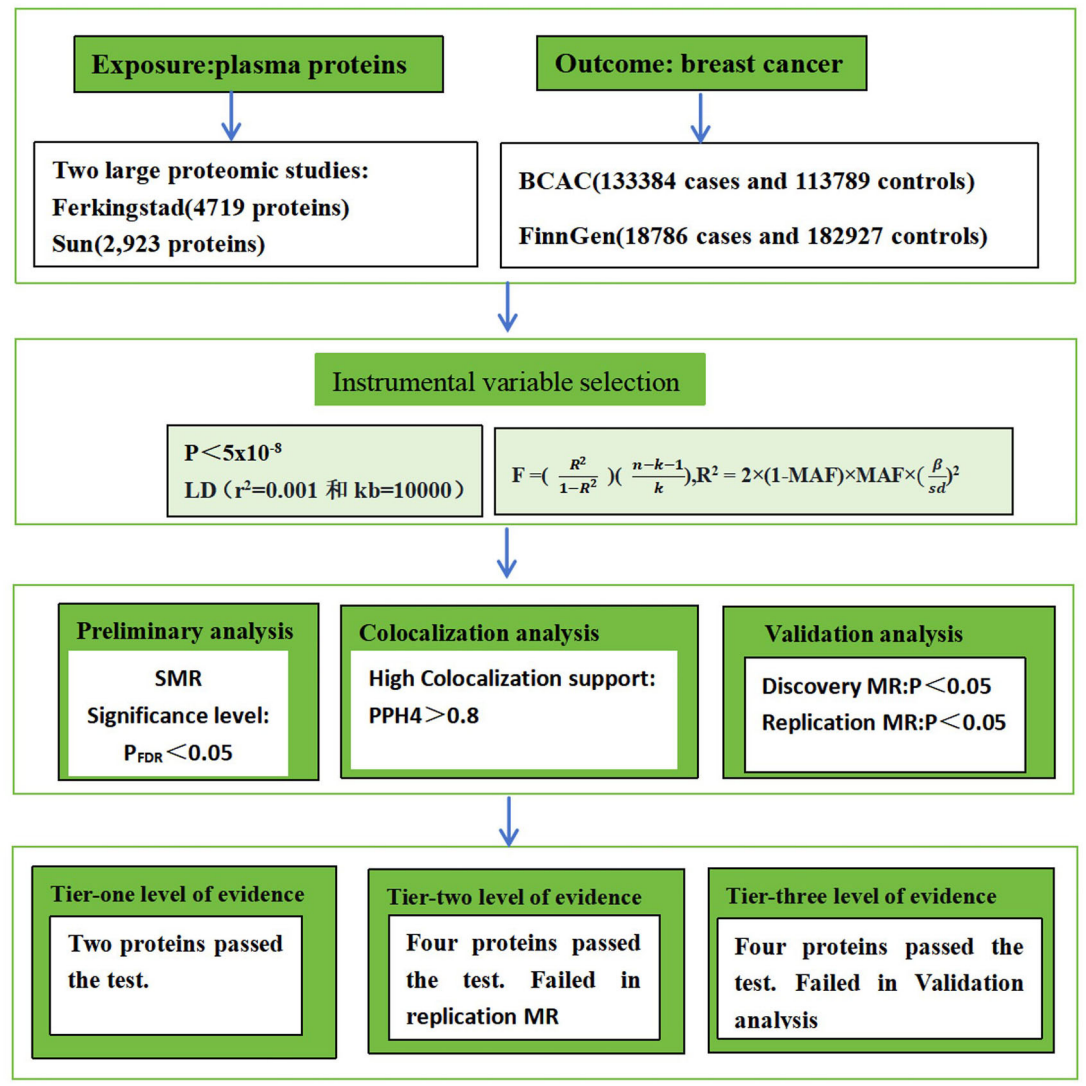
The workflow of study design. BCAC stands for Breast Cancer Association Consortium. LD stands for Linkage Disequilibrium. MR stands for Mendelian randomization. SMR stands for Summary-data-based MR.

Both preliminary and validation analyses were conducted in the present study. Protein quantitative trait locus (pQTL) data from two large-scale proteomic studies were used, and the SMR method was employed to evaluate the association between proteome and breast cancer. Positive findings from this initial assessment were subjected to Bayesian colocalization analysis. For proteins that met the criteria after colocalization analysis, the causal relationship with breast cancer was further validated using a two-stage MR framework, which included discovery and replication phases and was supplemented by sensitivity analyses. The MR analysis adhered rigorously to the following fundamental assumptions: (i) relevance assumption: single nucleotide polymorphisms (SNPs) are significantly associated with the exposure (protein expression levels); (ii) independence assumption: SNPs are independent of confounding factors, meaning they are not associated with variables that influence both the exposure and the disease outcome; (iii) exclusion restriction assumption: SNPs affect breast cancer risk exclusively through protein expression levels and not through other pathways (9) (**Figure 1**).

### 2.2 Proteomic data source

The pQTL data for the proteomic studies were obtained from research conducted by Ferkingstad et al. and Sun et al. The former study assessed the plasma protein levels in 35,559 Icelandic individuals using the SomaScan multiplex aptamer assay. They profiled 4,719 proteins and identified pQTL data for 18,084 protein quantitative trait loci (19). Sun et al. performed proteomic profiling of plasma samples from 54,219 participants in the UK Biobank using the antibody-based Olink Explore 3072 PEA technology. They profiled 2,923 distinct proteins and yielded pQTL data for 14,287 protein quantitative trait loci (20) (**Supplementary Table S1**).

### 2.3 Study population

The genetic data relevant to breast cancer were obtained from the Breast Cancer Association Consortium (BCAC) and the FinnGen Biobank. The BCAC consortium combined three datasets: iCOGS (38,349 cases and 37,818 controls), OncoArray (80,125 cases and 58,383 controls), and additional GWAS studies (14,910 cases and 17,588 controls), resulting in a total of 133,384 breast cancer cases and 113,789 controls (21). The FinnGen Biobank dataset includes 18,786 cases and 182,927 controls (22). In order to ensure the robustness of the study and minimize bias, the datasets used were exclusively derived from populations of European ancestry.

This study utilized publicly available databases, with all participant involvement ethically approved by their respective review boards and informed consent obtained from all subjects in the original studies (**Supplementary Table S2**).

### 2.4 Instrumental variable selection

The selection of genetic instruments for pQTL analysis adhered to the following criteria: first, genome-wide significant associations with a p-value of <5×10^−8^ were established, and SNPs significantly associated with any protein were extracted; an r² value of 0.001 and a distance of 10,000 kb were used to exclude SNPs in linkage disequilibrium (LD) (23, 24). Second, the F statistic ( $\text{F}=\left(\frac{R^{2}}{1-R^{2}}\right)\left(\;\frac{n-k-1}{k}\right),R^{2}\;=\;2\times \left(1-\text{MAF}\right)\times \text{MAF}\times (\frac{\beta }{sd}$ )^2^) was calculated to evaluate the strength of the association between SNPs and IVs, with an F > 10 indicating a sufficiently robust association to effectively mitigate bias from weak IVs (25). Third, SNPs within 1 Mb of the transcription start site of genes encoding proteins were classified as cis pQTL, whereas those outside this region were categorized as trans pQTL. Due to the considerable pleiotropy associated with trans pQTL, only cis pQTL were selected as IVs for this study (26).

### 2.5 Statistical analysis

The preliminary analysis utilized SMR, an extension of the MR concept, to investigate whether the effect sizes of SNPs on phenotypes are mediated by gene expression. This approach prioritizes GWAS hits for genes, facilitating subsequent functional investigations. These methodologies are applicable to various molecular quantitative trait loci data, including DNA methylation quantitative trait loci and pQTL. The present study employed SMR software with default settings via the command line for the analysis. The effect size (β) of variants indicated the direction of protein expression changes (16). The p-values from the results were adjusted using false discovery rate (FDR) correction, with associations possessing a PFDR < 0.05 considered statistically significant (27).

### 2.6 Colocalization analysis

In order to optimize outcomes, Bayesian colocalization analysis was used to evaluate whether protein expression and breast cancer are influenced by the same causal variant, thereby discerning confounding effects due to linkage LD. Bayesian colocalization analysis involved five hypotheses: H0, no significant association exists between protein expression and breast cancer with any SNP locus within a genomic region; H1, protein expression is significantly associated with SNP loci within a genomic region; H2, breast cancer is significantly associated with SNP loci within a genomic region; H3, protein expression, and breast cancer are significantly associated with SNP loci within a genomic region, but driven by different causal variants; and H4, protein expression and breast cancer are significantly associated with SNP loci within a genomic region, driven by the same causal variant (28). Colocalization analysis was conducted on all SNPs within ±500 kb of gene start sites, utilizing default parameters: P1 = 1×10^−4^ (prior probability of SNP association with protein), P2 = 1×10^−4^ (prior probability of SNP association with breast cancer), and P12 = 1×10^−5^ (prior probability of SNP association with both protein expression and breast cancer). Posterior probabilities were used to assess the support for each hypothesis, with a posterior probability of PP.H4 > 80% considered compelling evidence of colocalization (17).

### 2.7 Validation analysis

Validation analysis was conducted using a two-stage (discovery and replication) MR approach. In the Two-Sample MR analysis, the inverse variance weighted (IVW) method (29), the weighted median method (30), and the MR-Egger method (31) were employed as the primary analytical techniques. The IVW method, which has the highest statistical power, assumes the absence of an intercept term and that all genetic variants are valid IVs. On the other hand, the MR-Egger method accounts for the presence of an intercept term, though its testing efficiency may be less precise compared to the IVW method. As a complement to MR-Egger, the weighted median method allows for including some invalid variants, provided that at least half of them are valid IVs. In both the discovery and replication stages of MR, individual protein-level data (https://www.decode.com) were acquired, and separate two-sample MR analyses for breast cancer were conducted. A P value < 0.05 indicated a statistically significant association.

### 2.8 Sensitivity analysis

Cochran’s Q test was employed to evaluate heterogeneity among genetic variants. If the P-value of Cochran’s Q test was < 0.05, a random effects model was used for MR analysis; otherwise, a fixed effects model was applied (32). Additionally, the MR-Egger and MR-PRESSO methods were used to detect the presence of horizontal pleiotropy. By identifying and correcting for pleiotropy, MR-PRESSO can reduce bias caused by pleiotropy and provide more reliable causal effect estimates. Moreover, MR-PRESSO offers correction methods and evaluates the robustness of causal estimates through sensitivity analysis (33). We also used forest plots to assess the causal effect of each SNP and compared these with the causal estimates from the IVW and MR-Egger methods. A leave-one-out analysis was conducted by removing each SNP individually to evaluate whether a single variant drives the association between the exposure and outcome variables (34).

Ultimately, the selected proteins that met the criteria were categorized into three tiers based on the strength of evidence. Tier one included proteins meeting all standards in SMR, discovery MR, replication MR, and colocalization. Tier two included proteins meeting standards in SMR, discovery MR, and colocalization. Tier three included proteins meeting standards in SMR and colocalization.

The SMR tool version 0.1.3 (https://yanglab.westlake.edu.cn/software/SMR/#Overview) was utilized. All MR analyses were performed in R software version 4.3.1, using the Two Sample MR (version 0.5.9), MR-PRESSO (version 1.0), dplyr (version 1.1.3), circlize (version 0.4.15), stringr (version 1.5.0), ComplexHeatmap (version 2.15.4), and coloc (version 5.2.3) packages.

## 3 Results

### 3.1 Preliminary SMR and colocalization results

Following thorough IV processing, 7,981 cis pQTLs were utilized for SMR analysis, detecting significant associations with breast cancer susceptibility across 64 proteins (PFDR < 0.05) (**Supplementary Table S3**).

**Figure 2:**
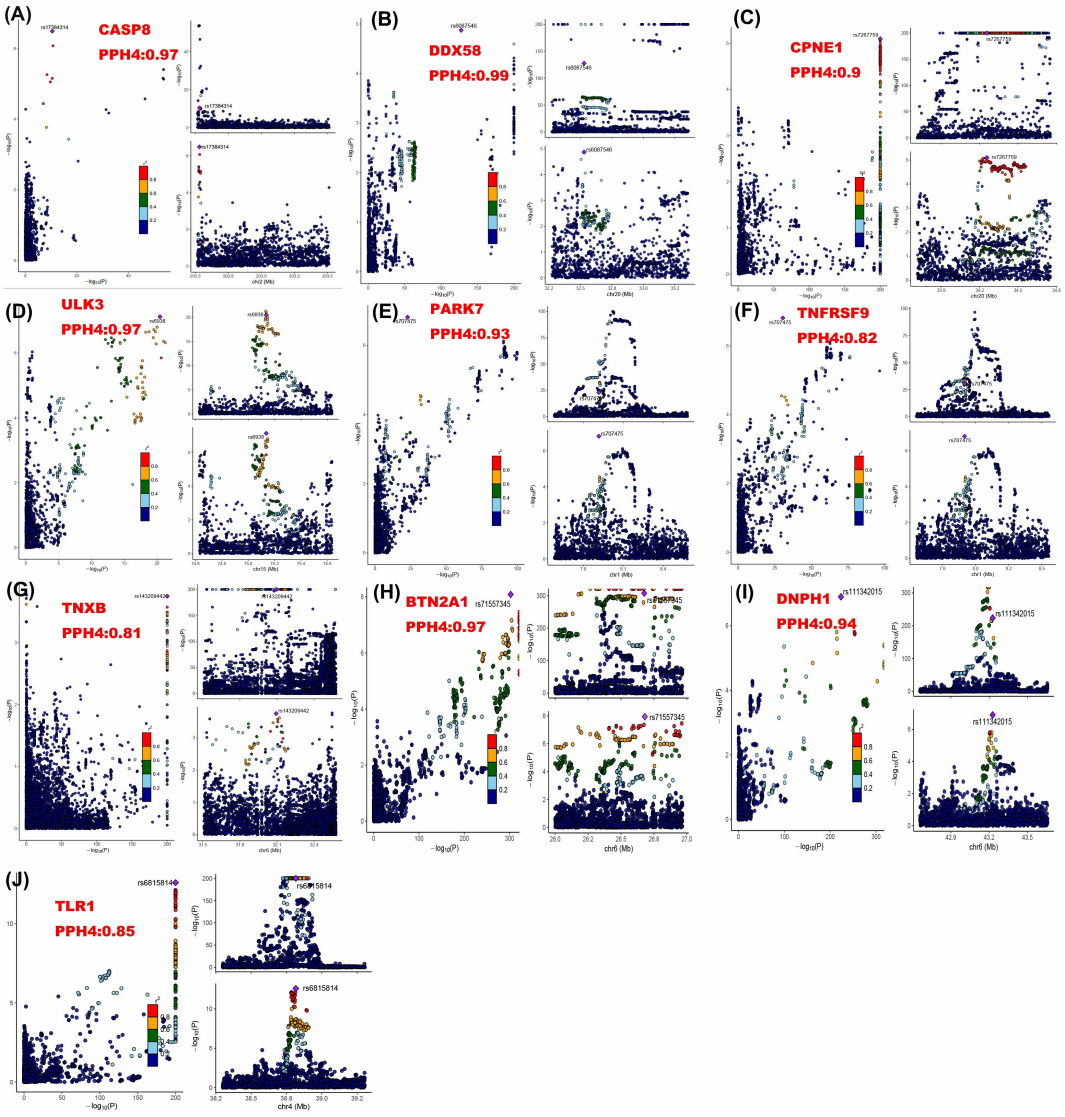
Regional association plots for colocalization analysis of ten proteins with breast cancer risk. The lead SNP is shown as a purple diamond. SNPs within ±500 kb of the protein quantitative trait locus were included; p12 = 1e-5, prior probability a SNP is associated with both protein and breast cancer.

Next, colocalization analysis was conducted separately for these proteins in relation to breast cancer. The results indicated that 10 proteins, CPNE1, TNXB, ULK3, CASP8, BTN2A1, PARK7, DNPH1, DDX58, TNFRSF9, and TLR1, had PP.H4 results of > 80% (**Table 1**; **Figure 2**).

**Table 1:** The summary of SMR results for the ten proteins that meet colocalization criteria with breast cancer.

Protein	Protein full name	SMR			Colocalization
		Beta P_FDR_ OR(95%CI)			PP.H4
CASP8	caspase 8	-0.15	8.34E-05	0.86(0.81-0.91)	0.97
DDX58	DExD/H-box helicase 58 (also known as RIG-I, Retinoic acid-Inducible Gene I)	-0.12	5.06E-04	0.89(0.85-0.93)	0.99
CPNE1	Copine 1	-0.04	5.34E-03	0.96(0.94-0.98)	0.90
ULK3	unc-51 like kinase 3	-0.37	0.01	0.69(0.59-0.81)	0.97
PARK7	Parkinsonism associated deglycase	-0.19	5.06E-04	0.83(0.77-0.90)	0.93
TNFRSF9	TNF receptor superfamily member 9	0.19	0.00	1.21(1.11-1.32)	0.82
TNXB	tenascin XB	0.04	0.04	1.04(1.02-1.07)	0.81
BTN2A1	butyrophilin subfamily 2 member A1	-0.14	3.06E-05	0.87(0.83-0.92)	0.97
DNPH1	2’-deoxynucleoside 5’-phosphate N-hydrolase 1	0.09	0.00	1.10(1.05-1.15)	0.94
TLR1	toll like receptor 1	0.10	2.56E-09	1.10(1.07-1.13)	0.85

SMR, Summary-data-based Mendelian randomization, PP.H4 values were all higher than 0.80 under priors (p12 = 1e−5) and windows (± 500kb).

### 3.2 Validation analysis

**Figure 3:**
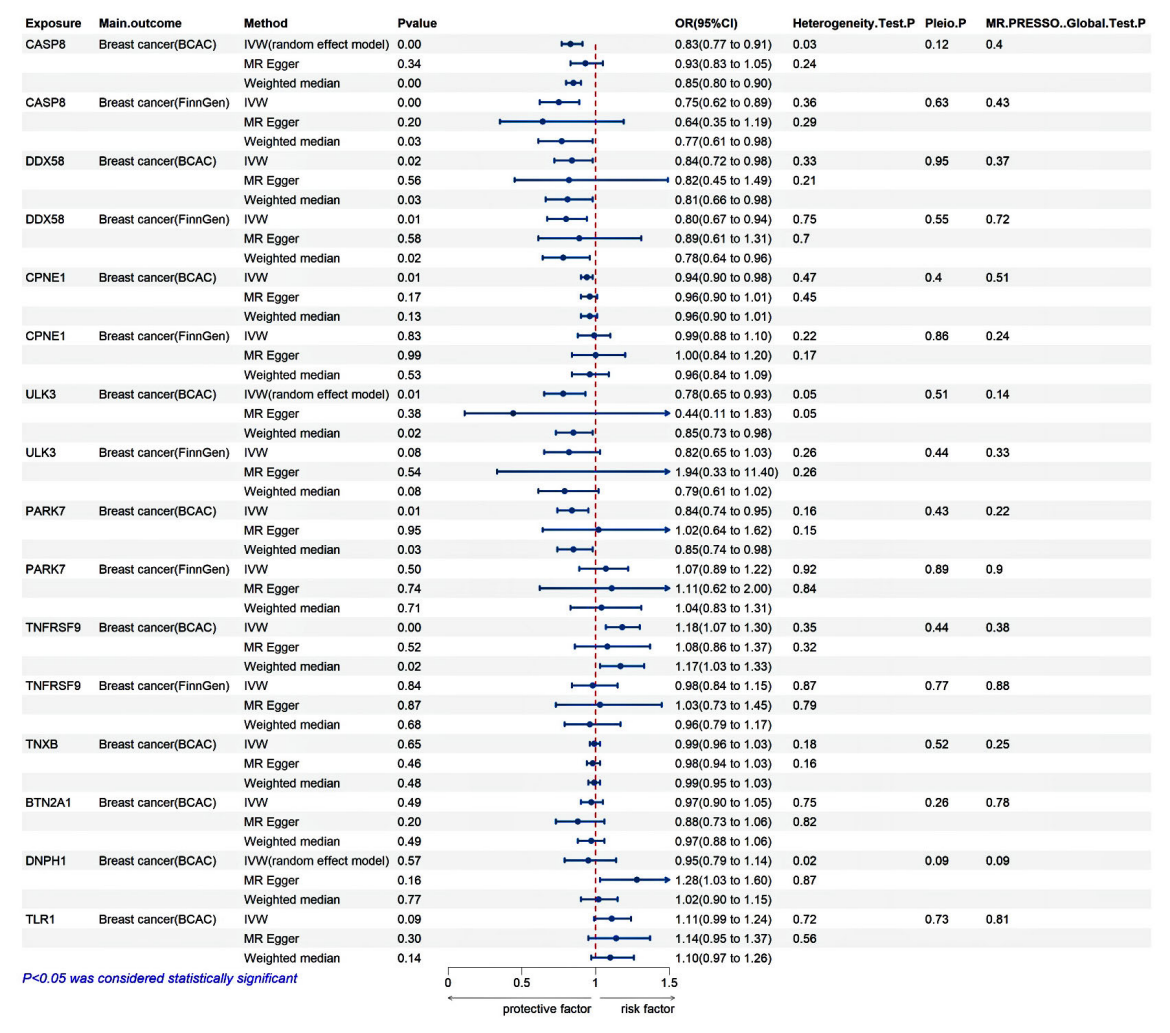
Validation analysis. The discovery MR is derived from the BCAC database, while the replication MR is derived from the FinnGen database.

In the discovery phase of MR, CPNE1 exhibited an inverse association with breast cancer risk (IVW: odds ratio (OR) = 0.94, 95% confidence interval (CI): 0.90-0.98, P = 0.005); ULK3 demonstrated a negative correlation with breast cancer risk (IVW: OR = 0.78, 95% CI: 0.65-0.93, P = 0.005); CASP8 revealed a negative correlation with breast cancer risk (IVW: OR = 0.83, 95% CI: 0.77-0.91, P = 2.74E-05); PARK7 manifested a negative correlation with breast cancer risk (IVW: OR = 0.84, 95% CI: 0.74-0.95, P = 0.005); DDX58 indicated an inverse association with breast cancer risk (IVW: OR = 0.84, 95% CI: 0.72-0.98, P = 0.023); and TNFRSF9 exhibited a positive association with breast cancer risk (IVW: OR = 1.18, 95% CI: 1.07-1.30, P =7.10E-04). However, TNXB, BTN2A1, DNPH1, and TLR1 had no significant correlation with breast cancer (P > 0.05 for all) (**Figure 3**; **Supplementary Table S4**).

In the replication stage of MR, CASP8 continued to show a negative correlation with breast cancer risk (IVW: OR = 0.75, 95% CI: 0.62-0.89, P = 0.001), and DDX58 maintained an inverse association with breast cancer risk (IVW: OR = 0.80, 95% CI: 0.67-0.94, P = 0.007). However, CPNE1, ULK3, PARK7, and TNFRSF9 did not replicate (P > 0.05 for all) (**Figure 3**; **Supplementary Table S4**).

### 3.3 Sensitivity analysis

Both MR-Egger regression and IVW methods detected heterogeneity in CASP8, DNPH1, and ULK3, prompting the use of a random effects model for MR analysis. Meanwhile, MR-Egger and MR-PRESSO methods detected no horizontal pleiotropy (**Figure 3**). To ensure the stability of the study results, the symmetrical distribution of SNPs in the funnel plot was confirmed. A leave-one-out analysis was performed to assess the influence of individual SNPs on the results, revealing no significant impact from any single SNP. Additionally, to gain a more comprehensive understanding of the data, scatter plots were generated to illustrate the causal relationships between proteins and breast cancer (**Supplementary Figure S1**-**S6**).

### 3.4 PPI and drug evaluation

**Figure 4:**
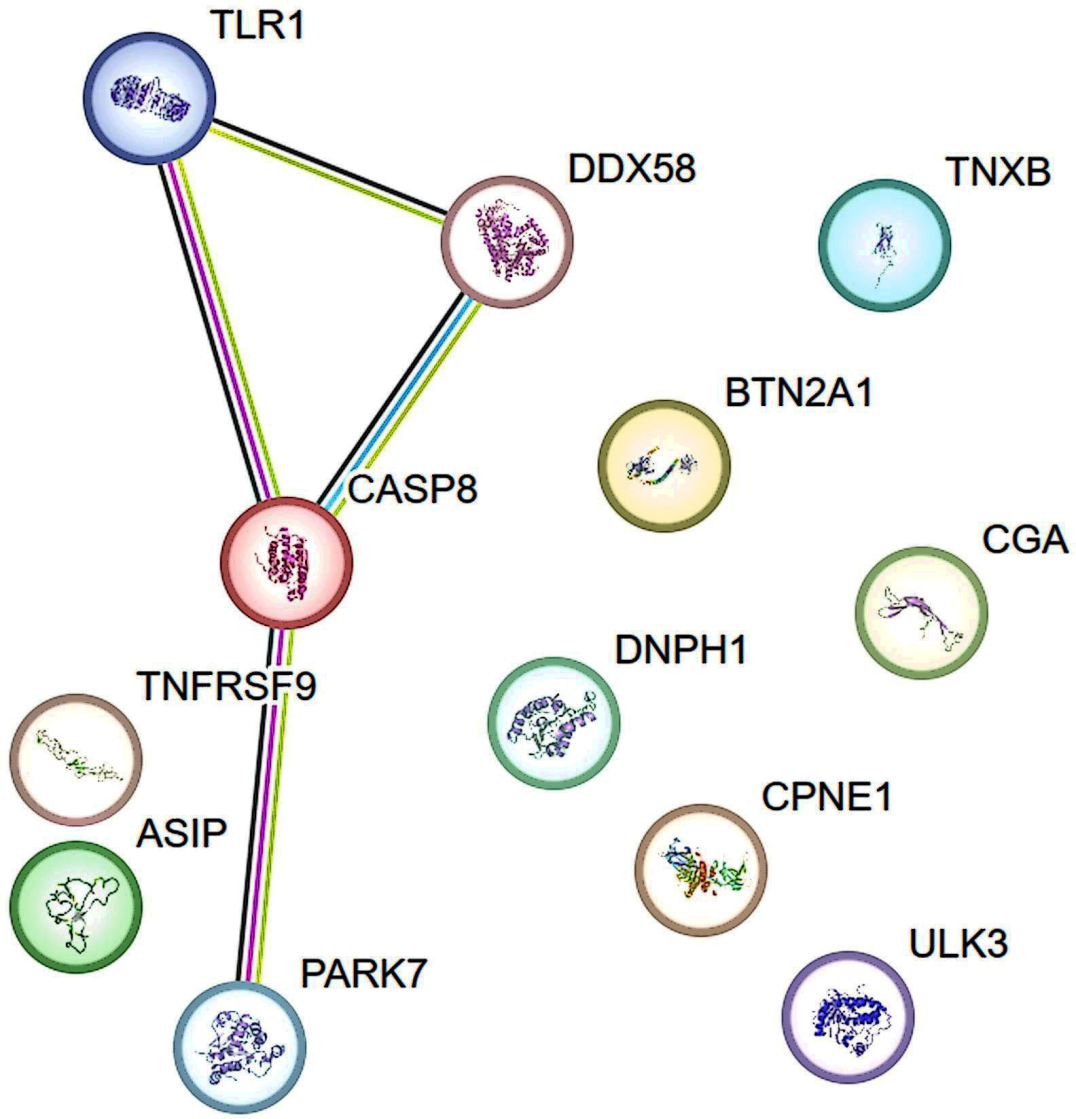
The Protein-protein interaction (PPI) network of proteins identified by proteome-wide Mendelian randomization. Lines represent interactions between proteins. Green line indicates gene neighborhood and predicted interaction; Blue line indicates known interaction from curated databases; Fuchsia line indicates known interaction that is experimentally determined. Black line indicates co-expression. Data information was from STRING database.

The STRING database was used to construct a protein-protein interaction (PPI) network to elucidate the connections among TLR1, CASP8, and DDX58 proteins, as well as the interactions involving CASP8 and PARK7 (**Figure 4**). Drug evaluation, conducted through platforms such as DGIdb4.0 (35) and DrugBank5.0 (36), identified CASP8, DDX58, CPNE1, ULK3, PARK7, and TNFRSF9 as notable targets for pharmacological exploration. Currently, therapeutic agents targeting CASP8 include bardoxolone, under investigation for lymphoma and solid tumor management; bryostatin 1, explored for HIV infection and Alzheimer’s disease intervention; AN-9, scrutinized for liver cancer, lung cancer, melanoma, and leukemia; trichostatin A; oleandrin, assessed for lung cancer therapy and chemotherapy-induced adverse effects. Pharmaceutical candidates targeting DDX58 include INARIGIVIR SOPROXIL, which is used as an immunomodulator and antiviral agent. Theophylline is a medication tailored for CPNE1 and used to mitigate symptoms associated with reversible airflow obstruction in conditions such as asthma, chronic obstructive pulmonary disease, and other pulmonary ailments. Fostamatinib, a spleen tyrosine kinase inhibitor, targets ULK3, providing therapeutic relief for chronic immune thrombocytopenia following alternative interventions. Therapeutic strategies aimed at PARK7 include copper, a transition metal present in various supplements and vitamins, and intravenous infusion solutions used in total parenteral nutrition. TNFRSF9 is targeted by Urelumab, currently under investigation for its efficacy against leukemia, multiple myeloma, malignant tumors, solid tumors, and B-cell non-Hodgkin’s lymphoma (**Supplementary Table S5**).

## 4 Discussion

The present study used publicly available large-sample pQTL and GWAS databases to analyze the causal relationship between 2,385 proteins and breast cancer, identifying 10 proteins associated with breast cancer risk. Among these, decreased levels of CASP8, DDX58, CPNE1, ULK3, PARK7, and BTN2A1 were found, alongside increased levels of TNFRSF9, TNXB, DNPH1, and TLR1. CASP8 and DDX58 exhibit the highest evidence strength, while CPNE1, ULK3, PARK7, and TNFRSF9 show secondary evidence strength. TNXB, BTN2A1, DNPH1, and TLR1 demonstrated the strength of the tertiary evidence. Druggability assessments prioritized six protein biomarkers already developed as drug targets for various chronic diseases or cancers, suggesting their potential repurposing as therapeutic targets for breast cancer.

Our study identified several candidate proteins previously linked to breast cancer, including CASP8, DDX58, CPNE1, PARK7, BTN2A1, TNFRSF9, TNXB, DNPH1, and TLR1, with CASP8 and DDX58 supported by the most robust evidence. CASP8, also known as caspase 8, is a critical initiator enzyme in the apoptosis pathway, which is vital in regulating programmed cell death (37, 38). Beyond its apoptotic function, CASP8 influences various cellular signaling pathways involved in inflammatory and immune responses (39). In oncology, CASP8 is recognized as a significant tumor suppressor gene, with aberrant expression or dysfunction linked to the onset, invasion, and metastasis. Experimental evidence indicates that CASP8 induces PD-L1 degradation by upregulating TNFAIP3 (A20) expression, and reduced CASP8 expression may predict sensitivity to anti-PD-L1/PD-1 immunotherapy (40). Previous studies have also suggested that the CASP8 D302H polymorphism decreases breast cancer risk associated with *BRCA1* and *BRCA2* mutations, delaying cancer onset (41). A preliminary study on Iranian breast cancer patients further reported significantly decreased CASP8 expression (42), which is consistent with our findings. Our preliminary SMR analysis and validation MR analysis both supported the protective role of elevated CASP8 levels against breast cancer risk (**Table 1**; **Figure 3**). Notably, a meta-analysis further substantiated the role of CASP8 in cancer susceptibility. This study assessed the association between CASP8 rs3834129 and rs1045485 polymorphisms with the risk of breast cancer and other malignancies, revealing that these polymorphisms significantly reduced the risk of breast cancer and several other cancers, particularly in Asian and Caucasian populations (43). These findings provide compelling evidence supporting the protective role of elevated CASP8 protein levels against breast cancer risk, underscoring the importance of CASP8 as a tumor suppressor gene in breast cancer. Additionally, the potential of CASP8 as a therapeutic target in other cancers, such as liver cancer, lung cancer, melanoma, and leukemia, has been explored, highlighting its broader applicability in cancer treatment (**Supplementary Table S5**). These studies emphasize the relevance of CASP8 in drug development, bolstering the case for considering CASP8 as a potential target in breast cancer therapy. While our results strongly support the protective effect of elevated CASP8 protein levels against breast cancer risk, the literature presents contrasting findings. For instance, a prospective observational study indicated that increased CASP8 levels might be associated with poorer prognosis in patients with metastatic breast cancer (44). This discrepancy could be due to differences in study populations, such as the distinction between metastatic patients and those with early-stage breast cancer, which may involve significant variations in disease progression and immune response. Alternatively, it may stem from differing research methodologies or analytical strategies. These divergences further underscore the importance of exploring the role of CASP8 across different cancer stages and subtypes.

DDX58, also known as RIG-I, is a critical intracellular pattern recognition receptor pivotal in immune responses. Cao et al. have shown that deficiencies in RIG-I contribute to chemotherapy resistance in triple-negative breast cancer by impeding apoptosis mediated through type I IFN signaling. They also found that patients with diminished DDX58 expression have lower rates of achieving pathological complete response and exhibit poorer prognosis (45). Additionally, studies focusing on innate immune strategies for activating breast cancer cells and the tumor microenvironment have shown that RIG-I activation within breast tumors enhances tumor-infiltrating lymphocytes while diminishing tumor growth and metastasis (46). These findings underscore the robust immunogenicity and therapeutic potential of RIG-I agonists when delivered to tumors, particularly in the context of less immunogenic breast cancers (46). Consistent with these observations, a previous study demonstrated that the active metabolite of tamoxifen (TAM), 4-hydroxytamoxifen (4-OH-TAM), regulates the expression of multiple genes, including the upregulation of DDX58 in estrogen receptor-positive (ER+) breast cancer MCF-7 cells. This research revealed that DDX58 and other genes were upregulated following 4-OH-TAM treatment, underscoring its role in both estrogen receptor-dependent and independent pathways (47). Our study indicates that lower levels of DDX58 protein were associated with an increased risk of breast cancer, which is consistent with previous foundational research (**Table 1**; **Figure 3**). Herein, we provided robust genetic evidence supporting the protective role of DDX58 against breast cancer risk. Currently, DDX58 is under investigation for its potential use as an immune modulator and antiviral agent, indicating its promise as a novel therapeutic target for breast cancer (**Supplementary Table S5**).

In the present study, CPNE1, PARK7, and TNFRSF9 were supported by secondary evidence strength. CPNE1 (Copine-1) is a calcium-binding protein with crucial roles in cellular signal transduction, adhesion, and apoptosis (48). Our research indicated that decreased circulating levels of CPNE1 are associated with an increased risk of breast cancer, which is consistent with findings by Ren et al. (14). However, multiple studies have also shown that CPNE1 promotes aerobic glycolysis and metastasis in triple-negative breast cancer (TNBC) through the PI3K/AKT/HIF-1α signaling pathway, thereby accelerating tumor progression (49). Additionally, other research has demonstrated that CPNE1 is overexpressed in TNBC tissues and cell lines, closely associated with tumor size, distant metastasis, and the survival rates of TNBC patients. CPNE1 also promotes tumorigenesis and radioresistance in TNBC cells by activating the AKT signaling pathway (50). Although most foundational studies suggest that CPNE1 has a pro-tumor role in cancer progression, our study and Ren’s research, employing MR, provide robust evidence from a causal perspective that CPNE1 may have a protective role in breast cancer. Our findings indicated a negative association between CPNE1 and breast cancer risk (OR: 0.94, 95% CI: 0.90-0.98), and Ren’s study yielded similar results (OR: 0.96, 95% CI: 0.94-0.98). This discovery suggests that a reduction in CPNE1 levels may increase the risk of breast cancer, which contradicts the tumor-promoting role of CPNE1 supported by conventional basic research. Therefore, while existing research predominantly focuses on the oncogenic role of CPNE1 in cancer, our and Ren’s study provide causal evidence through MR analysis, revealing the potential protective function of CPNE1. This causal insight offers a new perspective on CPNE1 as a potential therapeutic target in breast cancer and suggests that future research should further explore the dual mechanisms of CPNE1 to gain a more comprehensive understanding of its role in breast cancer progression.

PARK7, also known as DJ-1 protein, exhibits findings similar to those of CPNE1. PARK7 is widely expressed intracellularly and is involved in regulating cellular responses to oxidative stress, protecting mitochondrial function, maintaining cellular redox balance, and inhibiting apoptosis (51). The present study suggests that decreased circulating levels of PARK7 are associated with an increased risk of breast cancer (**Figure 3**), which is in line with findings by Wang et al. (52). In their retrospective study, Tsuchiya and colleagues demonstrated that DJ-1 protein expression in invasive ductal carcinoma (IDC) tissues was lower than in adjacent non-cancerous epithelial tissues despite higher mRNA levels. Among IDC patients, lower DJ-1 protein expression was significantly associated with shorter disease-free survival (P = 0.015) and overall survival (P = 0.020) (53). However, an observational study indicated that DJ-1 is upregulated in HR+ breast cancer and significantly correlates with poor prognosis (54). These discrepancies may stem from differences in the breast cancer molecular subtypes used in our analysis compared to traditional epidemiological studies, or they may underscore limitations in adjusting for confounding factors and reverse causation in traditional epidemiological research. In summary, the relationship between PARK7 and breast cancer risk remains inconclusive. PARK7 is supported by secondary evidence strength in our study, suggesting its potential as a therapeutic target for breast cancer. However, further experimental studies are needed to clarify the directionality of the associations between PARK7 and breast cancer.

TNFRSF9, also known as 4-1BB, is a protein that belongs to the tumor necrosis factor receptor superfamily and has a critical role in immune regulation, particularly in the activation and proliferation of T cells. When the 4-1BB receptor binds with its ligand, 4-1BBL, it triggers various signaling pathways, including AKT, NF-kB, and MAPK, promoting T cell proliferation, survival, and function (55). Currently, monoclonal antibodies targeting 4-1BB, such as urelumab and utomilumab, have been used in the treatment of B-cell non-Hodgkin lymphoma, lung cancer, breast cancer, soft tissue sarcoma, and other solid tumors (56). Our findings suggest that elevated levels of TNFRSF9 protein increase the risk of breast cancer (**Figure 3**), supported by secondary evidence strength. Our research enhances the genetic evidence linking TNFRSF9 elevation to an increased risk of breast cancer. Moreover, in their study, Harao et al. demonstrated that 4-1BB-enhanced expansion of CD8+ TILs can significantly promote the growth of these T cells within TNBC tumors. This approach can be used to identify immunogenic mutations within autologous TNBC tumor tissues. These findings underscore the potential application of 4-1BB in immunotherapy and offer a novel perspective on adoptive immunotherapy for TNBC (57). Our research further corroborates the role of TNFRSF9 in breast cancer and provides new genetic evidence supporting its potential as a therapeutic target.

ULK3 has emerged from our study as a novel prognostic biomarker for breast cancer. ULK3, or Unc-51 like autophagy activating kinase 3, primarily regulates the autophagy pathway within cells (58). Previous studies have identified the upregulation of ULK3 in squamous cell carcinoma of the skin and head and neck (59), and its potential as a prognostic biomarker in colon cancer (60, 61). However, there are currently no basic experimental research reports on the association between ULK3 and breast cancer. A preliminary SMR analysis conducted in the present study indicated a negative correlation between ULK3 and the risk of breast cancer (**Table 1**). This finding was confirmed by validation MR analyses (**Figure 3**). Notably, fostamatinib, a spleen tyrosine kinase inhibitor used for chronic immune thrombocytopenia after other treatments, targets ULK3 (**Supplementary Table S5**), which shows promise as a new therapeutic target for breast cancer treatment and as a prognostic biomarker.

In the present study, preliminary SMR analysis suggested potential causal relationships between TNXB, BTN2A1, DNPH1, CGA, TLR1, and breast cancer; however, these associations were not validated in the MR analysis, resulting in only tertiary evidence support, which indicates insufficient strength of evidence. Further research is necessary to confirm their associations with breast cancer.

This study has several strengths. Firstly, we systematically explored the relationship between plasma protein levels and breast cancer risk using a two-stage proteome-wide MR design. This approach benefits from a large sample size and comprehensive coverage. It also mitigates the risks of reverse causation and confounding biases. Secondly, our study included preliminary and validation studies, encompassing discovery and replication MR analyses, thereby providing robust evidence for our findings. Thirdly, we employed colocalization methods to minimize false positives arising from LD and horizontal pleiotropy. Additionally, our study samples were drawn from European populations, reducing potential biases related to racial differences in research outcomes. Fourthly, PPI and druggability assessments offer insights into the potential pathogenic roles of candidate proteins in breast cancer, aiding in the prioritization of druggable targets. Notably, proteins such as CASP8 and DDX58, which are already targeted for other diseases, exhibit promising potential as novel therapeutic targets for breast cancer.

However, the present study also has several limitations. Firstly, we did not investigate the relationship between circulating proteins and specific breast cancer subtypes due to the absence of cross-validated databases. This limitation underscores the necessity for future research to thoroughly explore the roles of these proteins across different breast cancer subtypes. Secondly, our study samples were exclusively from European populations, potentially limiting the generalizability of our findings to other ethnic groups. Further studies are needed to determine the applicability of these results across diverse racial populations. Thirdly, while we identified causal associations between relevant proteins and breast cancer, we could not conduct comprehensive biological experiments due to financial constraints. Future research could incorporate animal models and cell line experiments by addressing this gap to provide more robust evidence supporting our findings.

## 5 Conclusion

Using the MR combined colocalization method, we identified several plasma proteins associated with breast cancer risk, notably CASP8 and DDX58, which are promising targets for developing screening biomarkers and therapeutic drugs for breast cancer. Based on our findings, future experimental and clinical studies are essential to assess the efficacy and validate the potential of these candidate drugs.

## Data Availability

The original contributions presented in the study are included in the article/**Supplementary Material**, further inquiries can be directed to the corresponding author.
