# Supplementary material for: Identifying new biomarkers and potential therapeutic targets for breast cancer through the integration of human plasma proteomics: a Mendelian randomization study and colocalization analysis
Source: Front Endocrinol (Lausanne). 2024 Sep 16;15:1449668. doi: 10.3389/fendo.2024.1449668 (PMC11439655; doi:10.3389/fendo.2024.1449668)
Supplement: Supplementary file 1 [file DataSheet1.docx]

**Fig S1-S6 are visualizations of Leave-one-out plots, scatter plots, and funnel plots.**


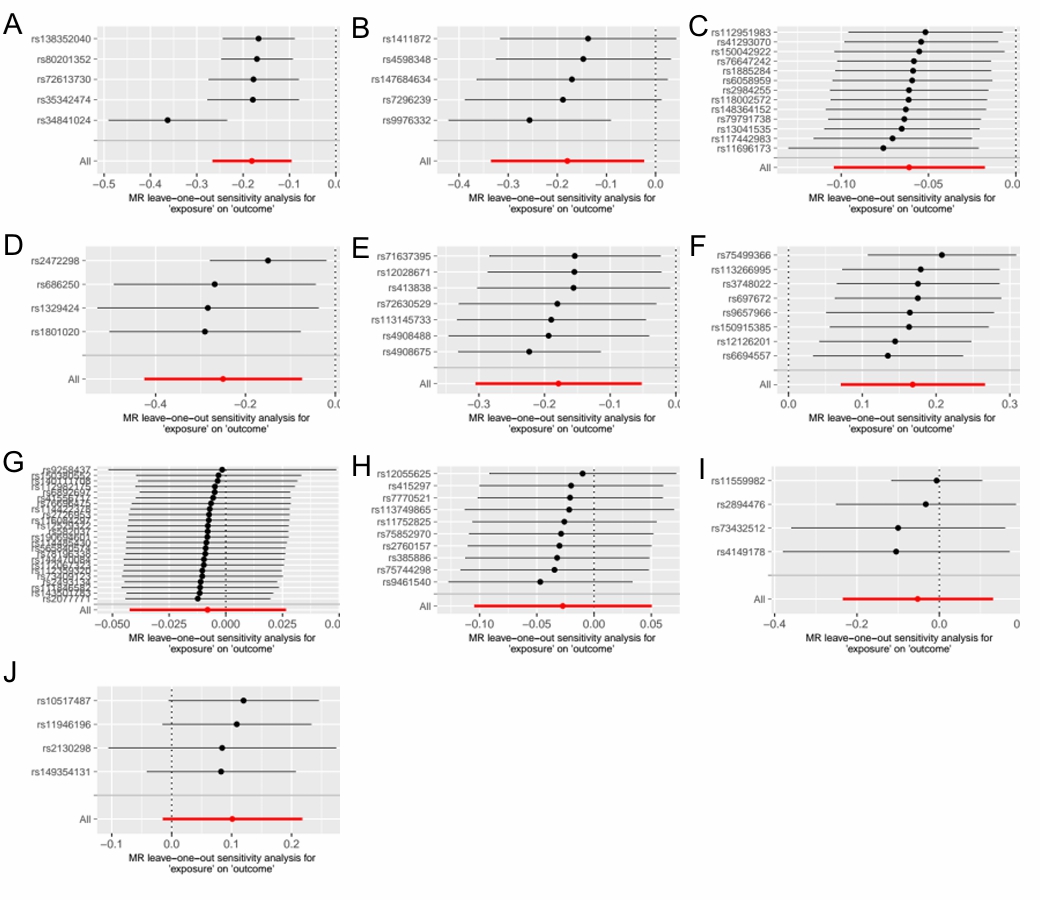


**Fig S1: Leave-one-out forest map of the causal relationships between ten proteins and breast cancer in the discovery phase of Mendelian randomization.**

**A:** Leave-one-out forest map of the causal relationship between CASP8 and breast cancer(BCAC). **B:**Leave-one-out forest map of the causal relationship between DDX58 and breast cancer(BCAC). **C:**Leave-one-out forest map of the causal relationship between CPNE1 and breast cancer(BCAC). **D:**Leave-one-out forest map of the causal relationship between ULK3 and breast cancer(BCAC). **E:**Leave-one-out forest map of the causal relationship between PARK7 and breast cancer(BCAC). **F:**Leave-one-out forest map of the causal relationship between TNFRSF9 and breast cancer(BCAC). **G:**Leave-one-out forest map of the causal relationship between TNXB and breast cancer(BCAC).

**H:** Leave-one-out forest map of the causal relationship between BTN2A1 and breast cancer(BCAC).

**I:** Leave-one-out forest map of the causal relationship between DNPH1 and breast cancer(BCAC).

**J:** Leave-one-out forest map of the causal relationship between TLR1 and breast cancer(BCAC).


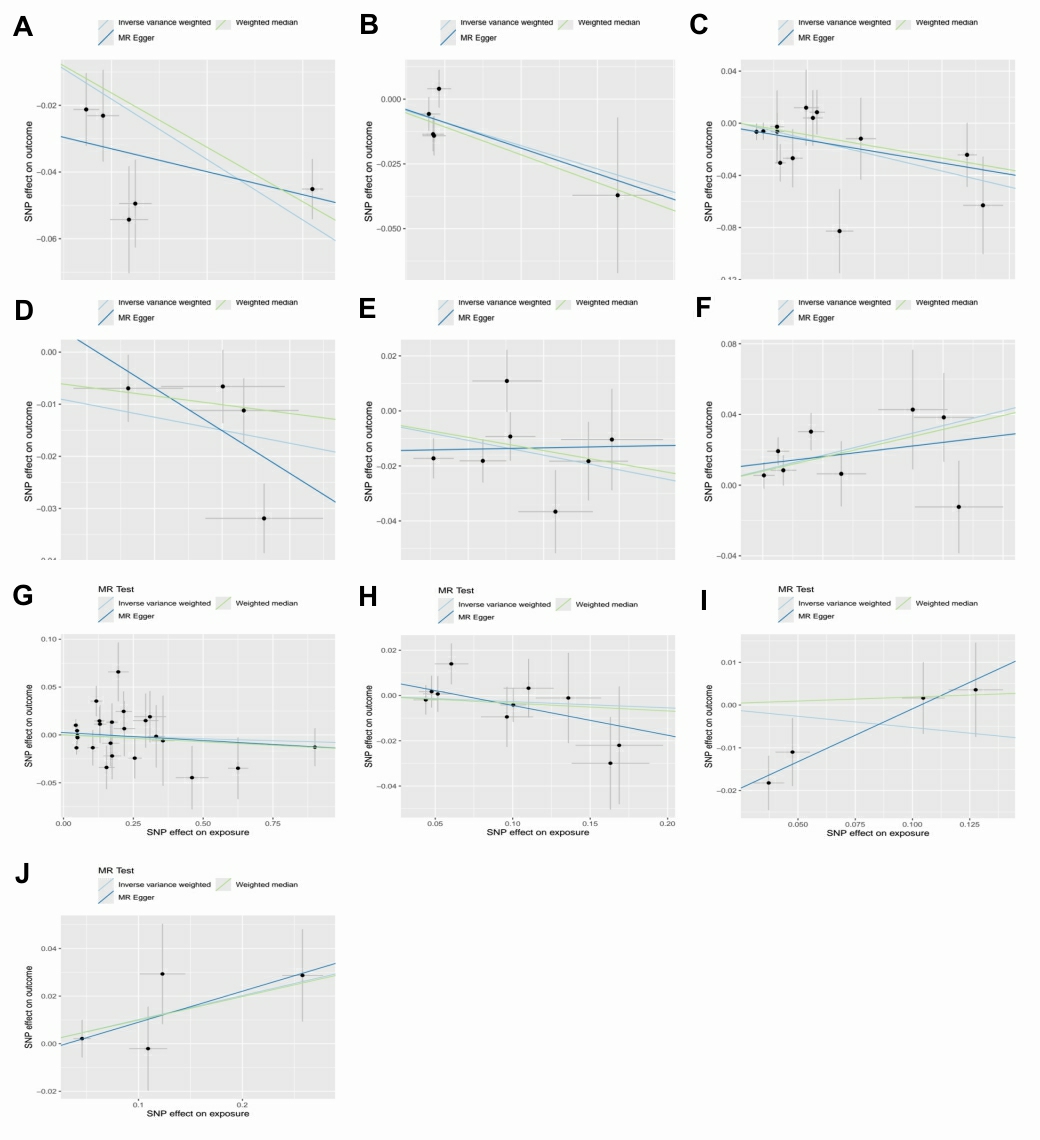


**Fig S2: Scatter plot of the causal relationships between ten proteins and breast cancer in the discovery phase of Mendelian randomization.**

**A:**Scatter plot of the causal relationship between CASP8 and breast cancer(BCAC). **B:**Scatter plot of the causal relationship between DDX58 and breast cancer(BCAC). **C:**Scatter plot of the causal relationship between CPNE1 and breast cancer(BCAC). **D:**Scatter plot of the causal relationship between ULK3 and breast cancer(BCAC). **E:**Scatter plot of the causal relationship between PARK7 and breast cancer(BCAC). **F:**Scatter plot of the causal relationship between TNFRSF9 and breast cancer(BCAC). **G:**Scatter plot of the causal relationship between TNXB and breast cancer(BCAC). **H:**Scatter plot of the causal relationship between BTN2A1 and breast cancer(BCAC). **I:**Scatter plot of the causal relationship between DNPH1 and breast cancer(BCAC). **J:**Scatter plot of the causal relationship between TLR1 and breast cancer(BCAC).


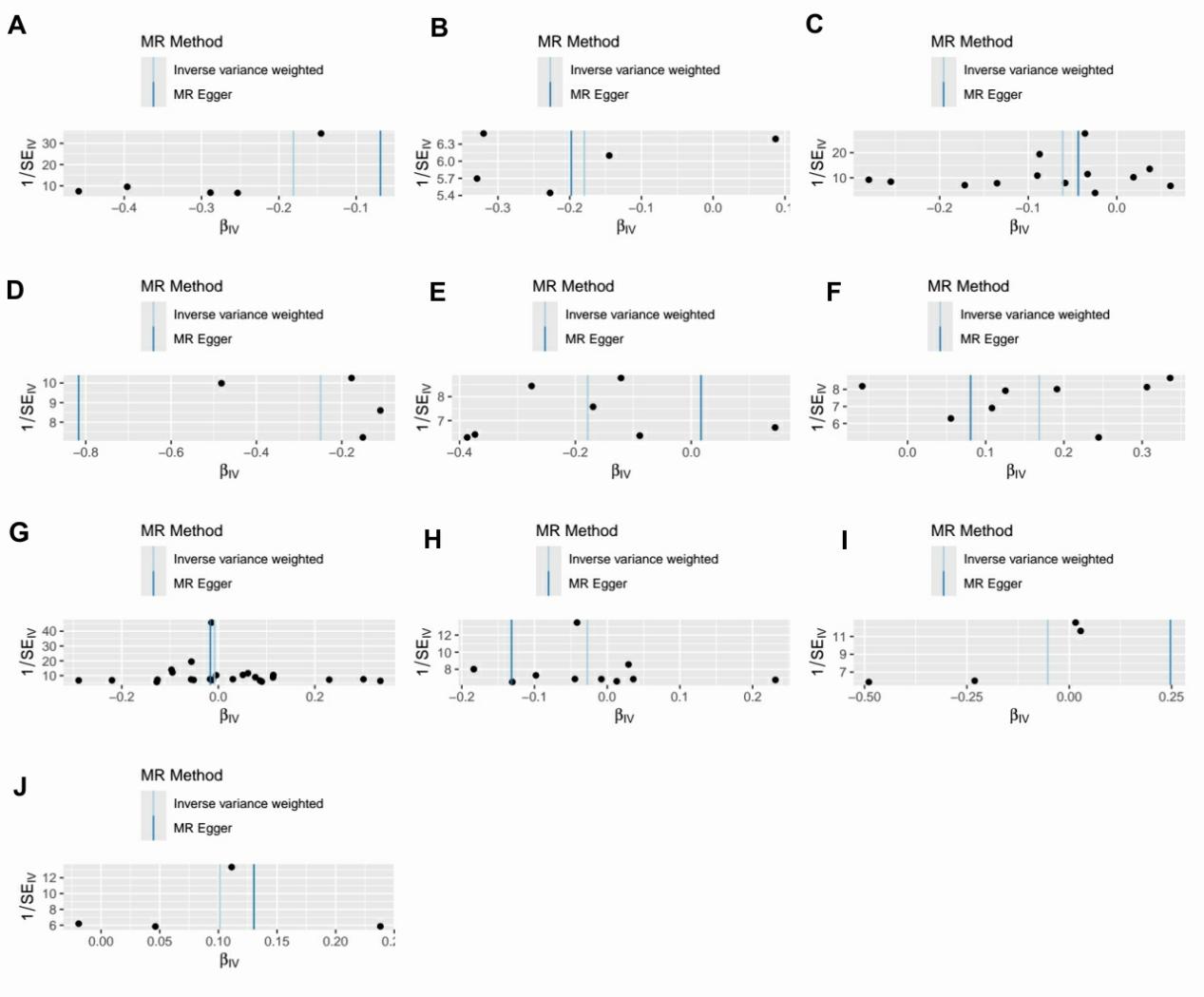


**Fig S3: Funnel plot of the causal relationships between ten proteins and breast cancer in the discovery phase of Mendelian randomization, primarily evaluated using the IVW method.**

**A:** Funnel plot of the causal relationship between CASP8 and breast cancer(BCAC). **B:**Funnel plot of the causal relationship between DDX58 and breast cancer(BCAC). **C:**Funnel plot of the causal relationship between CPNE1 and breast cancer(BCAC). **D:**Funnel plot of the causal relationship between ULK3 and breast cancer(BCAC). **E:**Funnel plot of the causal relationship between PARK7 and breast cancer(BCAC). **F:**Funnel plot of the causal relationship between TNFRSF9 and breast cancer(BCAC). **G:**Funnel plot of the causal relationship between TNXB and breast cancer(BCAC). **H:**Funnel plot of the causal relationship between BTN2A1 and breast cancer(BCAC). **I:**Funnel plot of the causal relationship between DNPH1 and breast cancer(BCAC). **J:**Funnel plot of the causal relationship between TLR1 and breast cancer(BCAC).

**
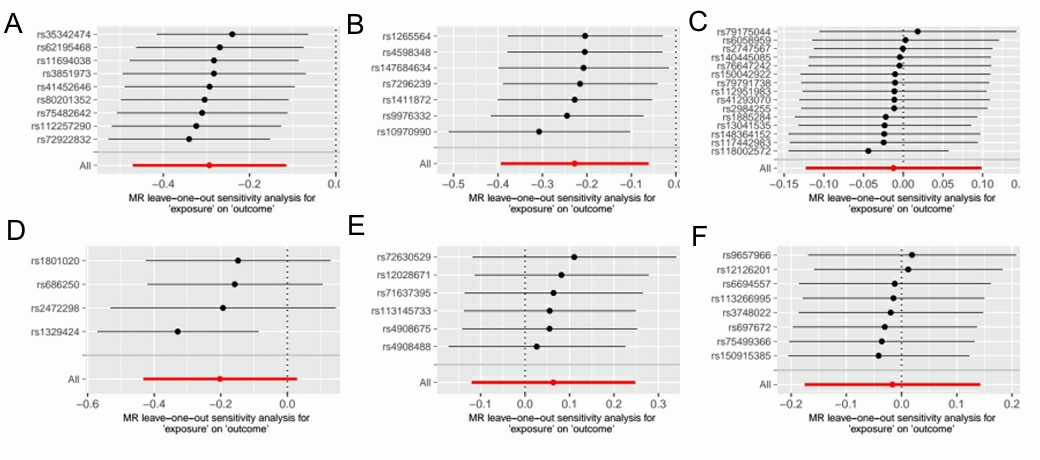
**

**Fig S4: Leave-one-out forest map of the causal relationships between six proteins and breast cancer in the replication phase of Mendelian randomization.**

**A:** Leave-one-out forest map of the causal relationship between CASP8 and breast cancer(FinnGen). **B:**Leave-one-out forest map of the causal relationship between DDX58 and breast cancer(FinnGen). **C:**Leave-one-out forest map of the causal relationship between CPNE1 and breast cancer(FinnGen). **D:**Leave-one-out forest map of the causal relationship between ULK3 and breast cancer(FinnGen). **E:**Leave-one-out forest map of the causal relationship between PARK7 and breast cancer(FinnGen). **F:**Leave-one-out forest map of the causal relationship between TNFRSF9 and breast cancer(FinnGen).


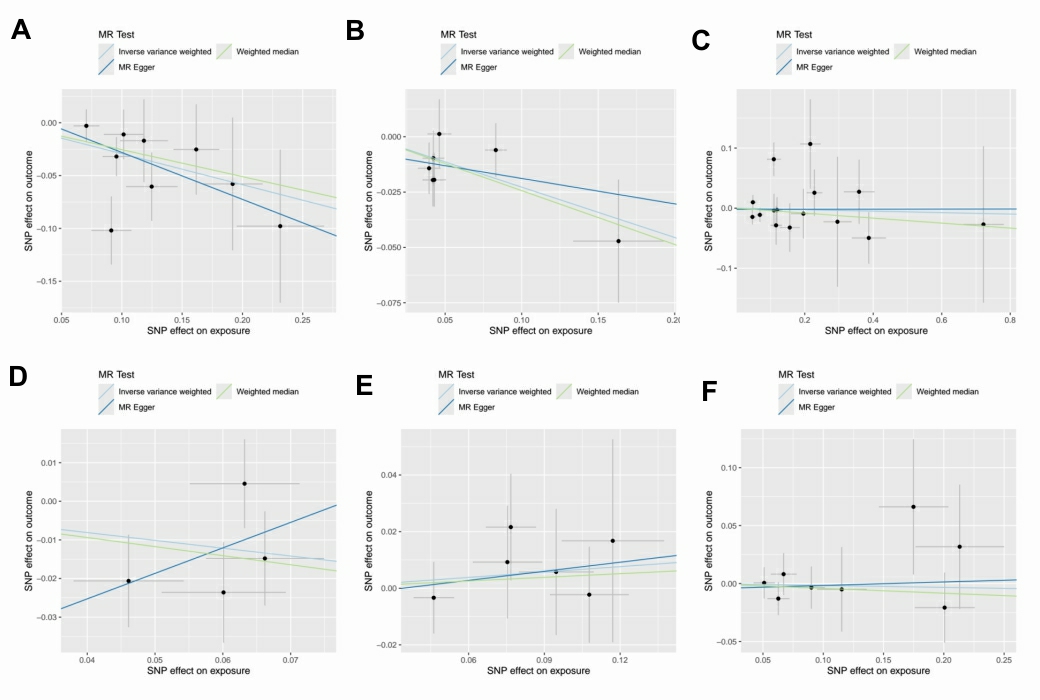


**Fig S5: Scatter plot of the causal relationships between six proteins and breast cancer in the replication phase of Mendelian randomization.**

**A:** Scatter plot of the causal relationship between CASP8 and breast cancer(FinnGen). **B:**Scatter plot of the causal relationship between DDX58 and breast cancer(FinnGen). **C:**Scatter plot of the causal relationship between CPNE1 and breast cancer(FinnGen). **D:**Scatter plot of the causal relationship between ULK3 and breast cancer(FinnGen). **E:**Scatter plot of the causal relationship between PARK7 and breast cancer(FinnGen). **F:**Scatter plot of the causal relationship between TNFRSF9 and breast cancer(FinnGen).


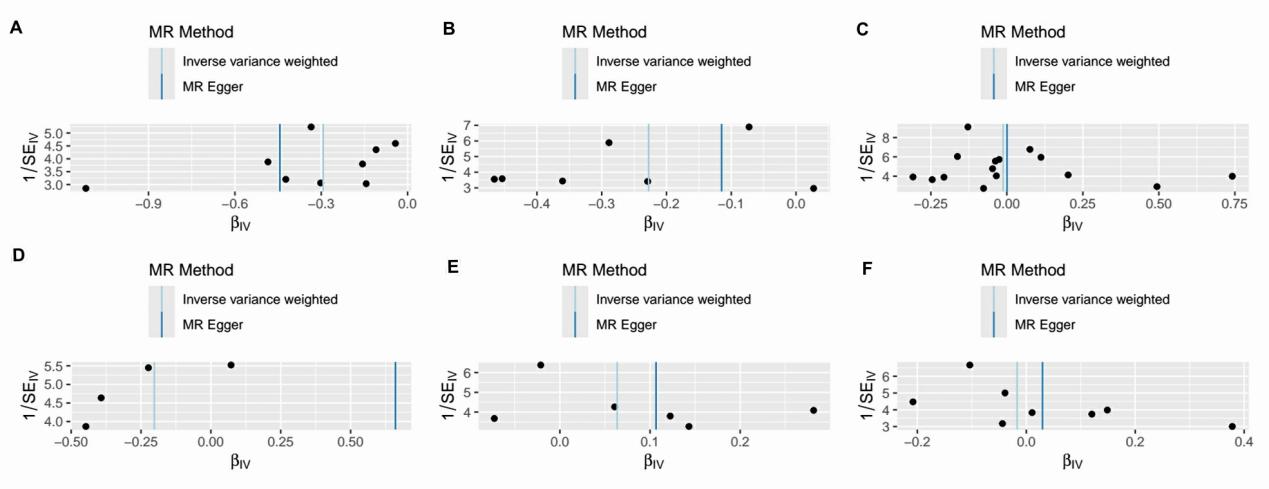


**Fig S6: Funnel plot of the causal relationships between six proteins and breast cancer in the replication phase of Mendelian randomization, primarily evaluated using the IVW method.**

**A:** Funnel plot of the causal relationship between CASP8 and breast cancer(FinnGen). **B:**Funnel plot of the causal relationship between DDX58 and breast cancer(FinnGen). **C:**Funnel plot of the causal relationship between CPNE1 and breast cancer(FinnGen). **D:**Funnel plot of the causal relationship between ULK3 and breast cancer(FinnGen). **E:**Funnel plot of the causal relationship between PARK7 and breast cancer(FinnGen). **F:**Funnel plot of the causal relationship between TNFRSF9 and breast cancer(FinnGen).
